# Tudor-SN, a component of stress granules, regulates growth under salt stress by modulating *GA20ox3* mRNA levels in *Arabidopsis*

**DOI:** 10.1093/jxb/eru334

**Published:** 2014-09-09

**Authors:** Chunxia Yan, Zongyun Yan, Yizheng Wang, Xiaoyuan Yan, Yuzhen Han

**Affiliations:** State Key Laboratory of Plant Physiology and Biochemistry, College of Biological Sciences, China Agricultural University, Beijing 100193, China

**Keywords:** *Arabidopsis*, *GA20ox3*, RNA immunoprecipitation, salt stress, stress granules, Tudor-SN.

## Abstract

The Tudor-SN protein (TSN) is universally expressed and highly conserved in eukaryotes. In *Arabidopsis*, TSN is reportedly involved in stress adaptation, but the mechanism involved in this adaptation is not understood. Here, we provide evidence that TSN regulates the mRNA levels of GA20ox3, a key enzyme for gibberellin (GA) biosynthesis. The levels of *GA20ox3* transcripts decreased in *TSN1*/*TSN2* RNA interference (RNAi) transgenic lines and increased in *TSN1* over-expression (OE) transgenic lines. The *TSN1* OE lines displayed phenotypes that may be attributed to the overproduction of GA. No obvious defects were observed in the RNAi transgenic lines under normal conditions, but under salt stress conditions these lines displayed slower growth than wild-type (WT) plants. Two mutants of *GA20ox3*, *ga20ox3-1* and *-2*, also showed slower growth under stress than WT plants. Moreover, a higher accumulation of *GA20ox3* transcripts was observed under salt stress. The results of a western blot analysis indicated that higher levels of TSN1 accumulated after salt treatment than under normal conditions. Subcellular localization studies showed that TSN1 was uniformly distributed in the cytoplasm under normal conditions but accumulated in small granules and co-localized with RBP47, a marker protein for stress granules (SGs), in response to salt stress. The results of RNA immunoprecipitation experiments indicated that TSN1 bound *GA20ox3* mRNA *in vivo*. On the basis of these findings, we conclude that TSN is a novel component of plant SGs that regulates growth under salt stress by modulating levels of *GA20ox3* mRNA.

## Summary

Tudor-SN protein accumulates in stress granules in response to salt stress in *Arabidopsis*. It binds *GA20ox3* mRNA *in vivo* and up-regulates *GA20ox3* levels to maintain plant growth under salt stress.

## Introduction

The Tudor-SN protein (TSN) is universally expressed and highly conserved in eukaryotes. It possesses four complete N-terminal staphylococcal nuclease (SNc) domains, a central Tudor domain, and a partial SNc domain at the C-terminus. TSN was first identified as a transcriptional co-activator in cultured animal cells (Tong *et al.*, 1995; Leverson *et al.*, 1998); it was subsequently shown to promote spliceosome assembly *in vitro*, to be a component of the RNA-induced silencing complex, and to exhibit nuclease activity (Caudy *et al*., 2003). TSN was reported to be targeted at multiple sites by caspases and metacaspases in animals and plants during apoptosis, which highlights the importance of TSN’s function in eukaryotes (Sundström *et al.*, 2009). Recently, Gao *et al*. (2010) reported that TSN interacts and co-localizes with G3BP in stress granules (SGs) under stress conditions in animals. In rice, TSN binds a variety of RNAs, including prolamine RNA, and associates with the cytoskeleton by interacting with other proteins (Sami-Subbu *et al.*, 2001). In pea, TSN interacts with the cytoskeleton (Abe *et al.*, 2003). Subsequent studies have shown that TSN participates in the localization of prolamine mRNAs in rice endosperm (Wang *et al.*, 2008). TSN is also involved in stress adaptation and RNA stabilization of its targets (Dit Frey *et al.*, 2010), and in the control of seed germination (Liu *et al.*, 2010).

Gibberellin (GA) is an important class of plant hormones that is necessary for normal plant growth and development. Bioactive GAs are synthesized through complex pathways and the rate-limiting steps are catalysed by GA 20-oxidase (GA20ox) and GA 3-oxidase (GA3ox) (Yamaguchi, 2008). There are five *GA20ox* genes in *Arabidopsis*, *GA20ox1*, *GA20ox2*, *GA20ox3*, *GA20ox4*, and *GA20ox5* (Hedden *et al.*, 2002). They convert C_20_-GA substrates to C_19_-GA products through successive oxidative reactions and are regulated by feedback from bioactive GAs (Huang *et al.*, 1998; Coles *et al*., 1999). Four of these genes, *GA20ox1*, *GA20ox2*, *GA20ox3*, and *GA20ox4*, possess GA20ox activity *in vitro* and catalyse all steps in the conversion of the C-20 intermediate GA_12_ to GA_9_, the immediate precursor of GA_4_ (Phillips *et al.*, 1995; Plackett *et al.*, 2012). However, GA20ox5 can only catalyse the first two steps (i.e. GA_12_ to GA_15_ and GA_15_ to GA_24_) in the reaction sequence, which also indicates partial GA20ox activity (Plackett *et al.*, 2012). The individual tissue expression patterns of the five GA20ox paralogues have been reported (Rieu *et al.*, 2008a). *GA20ox1* was the most highly expressed family member, especially in the stem and flower. *GA20ox2* was primarily expressed in 3- and 7-day-old seedlings. *GA20ox3* was the dominant gene expressed in dry seeds, imbibed seeds, and siliques. However, the levels of both *GA20ox4* and *GA20ox5* were low in most tissues. A loss-of-function mutation in *GA20ox1* reduced stem height, but the plants seemed to be developmentally normal otherwise (Koornneef and van der Veen, 1980). The antisense expression of *GA20ox2* and *GA20ox3* resulted in phenotypes similar to wild-type (WT) plants (Coles *et al*., 1999). However, overexpression of *GA20ox* resulted in GA-overproduction phenotypes in *Arabidopsis* (Huang *et al.*, 1998; Coles *et al*., 1999). Previous research also indicated that *GA20ox1* and *GA20ox2* demonstrate partial functional redundancy (Rieu *et al.*, 2008a). Recently, *GA20ox3* was shown to contribute to GA biosynthesis in multiple developmental processes and act redundantly with *GA20ox1* and *GA20ox2* (Plackett *et al.*, 2012). However, *GA20ox4* and *GA20ox5* played a minor role in growth and development; possibly, they promote flowering under long-day conditions (Plackett *et al.*, 2012).

SGs are a class of cytoplasmic foci that assemble in response to stress. SGs typically contain poly(A)^+^ mRNA, 40S ribosomal subunits, translation initiation factors, and RNA-binding proteins (Kedersha *et al*., 1999; Kedersha *et al*., 2002). Once formed, SGs serve as centres of mRNA triage, where mRNAs are targeted for storage, reinitiation, or degradation by processing bodies (PBs) (Kedersha *et al*., 2005). Recently, it was suggested that SG formation is required to allow optimal translation of stress-responsive mRNAs (Buchan and Parker, 2009; Vanderweyde *et al*., 2013); thus, SG formation plays an important role in enhancing resistance to stress (Kedersha and Anderson, 2007; Arimoto *et al*., 2008; Gareau *et al*., 2011). In plant cells, the cytoplasmic RNA granules were first identified in tomato cells as heat shock granules (HSGs; Nover *et al*., 1983; Nover *et al*., 1989). However, Weber *et al*. (2008) indicated that plant HSGs are not associated with mRNAs and are thus distinct from SGs in mammals. The same authors also revealed that plants do contain SGs that are conserved from mammals by detecting initiation factor eIF4E, poly(A)^+^ mRNA, RBP47, and UBP1 (Weber *et al*., 2008). In addition, a set of tandem CCCH zinc finger proteins, TZF1, -4, -5, and -6, were reported to be localized to SGs and PBs (Pomeranz *et al*., 2010; Bogamuwa and Jang, 2013). However, the functions of SGs in plants are still unknown.

Although TSN is reportedly involved in stress adaptation in *Arabidopsis*, the mechanism for this adaptation is not understood. In this study, we confirmed that TSN regulates *GA20ox3* mRNA levels and that GA20ox3 is necessary for plant growth under salt stress conditions. Furthermore, the analysis of transiently co-expressed TSN1 and RBP47, a marker protein for SGs, indicated that TSN is localized to stress granules in response to salt stress. On the basis of RNA immunoprecipitation (RIP) experiments, we also showed that TSN1 binds *GA20ox3* mRNA *in vivo*. Thus, we conclude that TSN is a novel component of SGs and regulates the growth of *Arabidopsis* under salt stress by modulating *GA20ox3* mRNA levels.

## Materials and methods

### Plant materials and growth conditions

*Arabidopsis* ecotype Columbia plants were used as the WT plants. The mutant of *ga20ox3-1* was provided by the Plant Science Department of Rothamsted Research. The *ga20ox3-2* mutant (CS91343) was obtained from the *Arabidopsis* Biological Resource Center. Seeds were surface sterilized with 6.4% sodium hypochlorite solution for 15min, washed at least three times with autoclaved water, and germinated on MS medium supplemented with sucrose (3%) and agar (1%) at pH 5.7. Plants were grown in horticultural soil in a growth chamber (19–22 °C, 16h light/8h dark photoperiod, 80% relative humidity).

### Construction of pSuper-TSN1 and pFGC–*TSN1/TSN2* RNAi vectors

The full-length of *TSN1* (*AT5G61780*) was amplified from *Arabidopsis* genomic cDNA using the primers *OE*-LP (5′-AGGTC TAGAATGGCGACTGGGGCAGCAACT-3') and *OE*-RP (5′-AT TGGTACCTTACCCGCGACCCGG TTTCCT GAC-3′). *TSN1* amplification products and pSuper 1300 plasmid (Guo *et al*., 2013) were digested with *Xba*I and *Kpn*I. Both digestion products were linked to generate *pSuper-TSN1.*

We selected a 359-bp fragment (+2,417–+2,775) to obtain the *TSN1/TSN2* (*AT5G07350*) double gene silencing mutant for the *pFGC-TSN1/TSN2* RNAi vector. *TSN1* and *TSN2* share nearly 85% homology in this zone and there is generally low homology between *TSN1/TSN2* and other genes in *Arabidopsis*. The fragment was amplified using the primers RNAi-sense-LP (5′-CCGCTC GAGC AGTTCAAT CT CC AGAG-3′) and RNAi-sense-RP (5′-GCCATTTAAATCTGAAGCATTGCTGC ATTG-3′); the fragment was then cloned into the upstream region of the GUS sequence of the pFGC1008 vector (Gong *et al*., 2002) after *Xho*I-*Swa*I digestion. The 359-bp fragment was amplified again using primers RNAi-anti-LP (5′-GGACTAG TGAGCA GTTCAA TCTC CAGAG-3′) and RNAi-anti-RP (5′-CGGGATTCCTGAAGCATTGCTGCATTG-3′). The fragment was then cloned into the downstream region of the GUS sequence of the pFGC1008 vector after *Spe*I-*Bam*HI digestion. This construct was named pFGC-*TSN1/TSN2* RNAi.

### Plant transformation and selection

*Agrobacterium tumefaciens* GV3101 (from State Key Laboratory of Plant Physiology and Biochemistry, China Agricultural University) was transformed with constructs of the *pSuper-TSN1* vector and pFGC-*TSN1/TSN2* RNAi vector by electroporation. *Arabidopsis* plants were transformed via *Agrobacterium tumefaciens* by the floral dip method as described previously (Clough and Bent, 1998). Putative transformants were selected on MS plates supplemented with 25 µg ml^–1^ hygromycin B.

### Quantitative and semi-quantitative RT-PCR analysis

Total RNA was isolated according to a previously described method (Oñate-Sánchez and Vicente-Carbajosa, 2008). A total of 7 µl of RNA was reverse-transcribed using oligo(dT) primer and M-MLV reverse transcriptase (TaKaRa, Japan). The quantity of cDNA was determined by the Nanodrop-1000. Each cDNA sample was diluted ten-fold with ddH_2_O. Quantitative RT-PCR was performed with 20 µl of reaction mixture that contained 4 µl cDNA, 0.4 µl each of forward and reverse primers, 0.4 µl Rox Reference Dye II (50X), and 10 µl SYBR Premix Ex Taq (TaKaRa, Japan). The reaction was completed on an ABI 7500 Real-Time PCR System. PCR conditions were 95 °C for 30 s, followed by 40 cycles of 5 s at 95 °C and 35 s at 60 °C. Fusion curves were characterized by 0.5 °C/cycles ramping from 60 °C–95°C. Three biological replicates were performed for each experiment, incorporating three technical replicates of each reaction. The relative transcript levels of synthesis were calculated using the 2^–ΔΔC^_T_ method (Schmittgen and Livak, 2008). Data were normalized with respect to *At4G34270*. The following primers were used for quantitative RT-PCR: *TSN1*-qLP (5′-GCTGGCCTGGCAAAAATG-3′) and *TSN1-*qRP (5′-AGAA TATGAGCTTCGGGAATCCT-3′); *TSN2*-qLP (5′-GAGG TGGT CGGTT CTATGTTC-3′) and *TSN2*-qRP (5′-CCGATAATGGGA GCGTCTTT-3′); *GA20ox1-*qLP (5′-GCATCAGCGAGGAGCTTA TT-3′) and *GA20ox1-*qRP (5′-CCAAC ACTC TCACCGGATTT-3′); *GA20ox2-*qLP (5′-AGCAGTTTGGGAAGGTGTATC-3′) and *GA 20ox2-*qRP (5′-CCTCGGAAA TAGTCTCGGTTTAC-3′); *GA20 ox3-*qLP (5′-TGGG CGAT GGATACGAAGA-3′) and *GA20ox3-*qRP (5′-CATGGCC TCCGC GTAT TC-3′); and *At4G34270*-qLP (5′-GTGAAAACTGTTGGAGAGAAGCAA-3′) and *At4G 34270*-qRP (5′-TCAACTGGATACCCTTTCGCA-3′). For semi-quantitative PCR, 1 µl cDNA was used per reaction. PCR reactions (25 µl) were performed using Taq DNA polymerase (TaKaRa, Japan) for 30 cycles. The following primers were used for semi-quantitative PCR: *TSN1*-LP (5′-AAGGAGACAATCAACACAAGA-3′) and *TSN1*-RP (5′-GA CAGCG ATGAGAGTTACAAC-3′); *T SN2*-LP (5′-AATGGA AGTGTT GTTGA GACAG-3′) and *TS N2*-RP (5′-ATTCCAATTCTCGACTTGCG-3′); *GA20ox3*-LP (5′-ATCA GCACTCGCACCACAT-3′) and *GA20ox3*-RP (5′-AGCGT GAGGGTT AGGA GG-3′); and *Actin2*-LP (5′-CACTGTGCCAAT CTACGAGGGT-3′) and *Actin2*-RP (5′-GCTG GAATGTGCTGAG GGAAG-3′).

### Seed germination analysis and root elongation measurements

Seeds were surface-sterilized as described above and sown on MS agar medium. Both experimental and control lines were grown on each plate (100 seeds per genotype). After stratification for 2 d at 4 °C, seeds were grown in a growth chamber in the absence of stress (normal condition). Germination percentages were scored every 12h. After germination was complete, the synchronized growth seedlings (same root length) were transferred to MS medium containing NaCl or mannitol to determine the root length under stress conditions. The root lengths of 6-day-old seedlings were measured with a ruler; thirty seedlings per genotype were measured. For fresh weight determination, the weight of at least 30 seedlings was measured. For GA response experiments, seeds were germinated on MS agar medium for 2 d and then the synchronized growth seedlings were transferred to MS medium containing 150mM NaCl plus 10 µM or 100 µM GA_3_ and grown for 6 d. In the relative root growth test, the relative values were determined as the ratio of root length under stress to root length under normal condition following a previously described method (Ezaki *et al*., 2007).

### Flowering time and plant height statistics

*TSN1* overexpression (OE) transgenic lines and WT plants were grown in alternating periods of 16h of light and 8h of dark. The flowering time for each genotype was recorded and the number of rosette leaves was counted; 40 plants in each line were measured. The stem length was measured in 35-day-old plants.

### Subcellular localization of TSN1 fused to green fluorescence protein (GFP) and RBP47 fused to red fluorescence protein (RFP)

The full-length *TSN1* coding sequence with the stop codon replaced by six Gly codons was amplified from *Arabidopsis* genomic cDNA. We used the following primers for amplication: *TSN1-OE*-LP (5′-AGGGGATCCATGGCGACTGGGGCAGCAACT-3′) and *TSN1-OE*-RP (5′-ATTGG TACCG GAGGAG GTGGTGG TGGTCCCG CGACCCG GTTTC CTGAC-3′). *TSN1* amplification products and pBI121–GFP plasmid (a gift from Qijun Chen, China Agricultural University) were digested with *Bam*HI and *Kpn*I. Next, *TSN1* amplification products were cloned into the pBI121–GFP vector, in which GFP was fused to the C-terminus of TSN1. The construct, which was named pBI121–*TSN1*–GFP, was transformed into *Arabidopsis* plants to generate stable expression lines. Nine homozygous transgenic lines which displayed GFP fluorescence were obtained. These lines showed phenotypes similar to that of OE transgenic lines. The GFP fluorescence of the roots of 5-day-old seedlings was observed using a Zeiss 510 META confocal laser scanning microscope with excitation wavelength 488nm and emission wavelengths 505–530nm. To analyse transient expression, *Arabidopsis* mesophyll protoplasts from rosette leaves of 4-week-old WT plants were prepared as described by Kim and Somers (2010). The RFP–RBP47 vector was generously provided by Markus Fauth (Weber *et al*., 2008). The TSN1–GFP and RFP–RBP47 vectors were co-transformed into *Arabidopsis* protoplasts and incubated for 20h. The cells were then either kept under control conditions or subjected to 150mM NaCl. Co-localization analyses were performed using a Zeiss 510 META confocal laser scanning microscope with excitation wavelength 488nm and emission wavelengths 505–530nm for GFP and excitation wavelength 543nm and emission wavelengths 560–615nm for RFP.

### Western blotting

Total proteins of homozygous *GFP1* and *GFP2* lines, which displayed the phenotypes similar to the *OE*5 line, were extracted in 20mM Tris-HCl, pH 8.0, 5mM EDTA, 1mM PMSF, 0.05% SDS, 5mM EGTA, and 10mM DTT. After sedimentation at 4 °C for 1h, the solution was centrifuged at 12 000×g for 30min. The supernatants were then used for protein quantification in a Microplate system as described previously (Dreher *et al*., 2005). Total proteins were electrophoresed in 10% SDS-PAGE and the gels were transferred onto polyvinylidene fluoride microporous membranes. The membranes were treated with anti-GFP antibodies (Roche, Germany) diluted 1000 fold for 1h at 4 °C, followed by treatment with secondary antibodies (Goat anti-Mouse IgG-HRP, Abmart, China) for 1h.

### RNA immunoprecipitation

We based our RNA immunoprecipitation experiments on previously described methods (Gilbert and Svejstrup, 2006; Terzi and Simpson, 2009). The 7-day-old seedlings of TSN1–GFP transgenic lines and WT plants were harvested, washed 4 times in cold, sterile water for 1min, and vacuum-infiltrated in 0.5% formaldehyde for 2min. The seedlings were then released from the vacuum and vacuum-infiltration was reapplied for 8min. Fixation was stopped using 2M glycine under vacuum for 1min. The vacuum was released and vacuum-infiltration was reapplied for 4min. Total proteins of these seedlings were extracted as described above. The protein extracted buffer was supplemented with 40U µl^–1^ RNase inhibitor.

For immunoprecipitation reactions, 40 µl of protein A agarose beads (Abmart, China) were washed three times in 1ml of binding/washing buffer (20mM Tris-HCl, pH 8.0, 150mM NaCl, 2mM EDTA, 1% Triton X-100, 0.1% SDS, 1mM PMSF). To decorate the beads with anti-GFP antibodies, 0.1 µl of anti-GFP antibodies was added and diluted in 100 µl of binding/washing buffer. The beads were then incubated with anti-GFP antibodies for at least 3h at 4 °C under rotation. Next, the anti-GFP-decorated beads were washed three times in 1ml of binding/washing buffer for 5min at 4 °C under rotation. The beads were stored at 4 °C in 100 µl of binding/washing buffer before the immunoprecipitation reactions.

For the immunoprecipitation of RNA–protein complexes, the binding/washing buffer was removed from the beads decorated with anti-GFP antibodies. 60 µl of total protein were diluted ten-fold with chip dilution buffer (16.7mM Tris-HCl, pH 8.0, 1.1% Triton X-100, 1.2mM EDTA, 167mM NaCl) that contained 6 µl RNase inhibitor and then added into beads. Antigen–antibody complex formation was performed overnight at 4 °C on a rotator. Supernatants were then removed and the beads were washed three times in 1ml of binding/washing buffer. Then the beads were diluted with 1ml of binding/washing buffer and transferred to fresh Eppendorf tubes and centrifuged at 2000rpm for 1min. The binding/washing buffer was removed and 50 µl RIP dilution buffer (100mM Tris-HCl, pH 8.0, 10mM EDTA, 1% SDS) was added. The samples were rotated for 10min at room temperature. The supernatants were transferred to fresh Eppendorf tubes and the beads were again eluted with 50 µl RIP dilution buffer at 65 °C. Finally, the supernatants were combined with the first supernatant samples. These samples were then treated with 1 µl 20mg ml^–1^ proteinase K for 1h at 65 °C. For input preparation, 100 µl RIP dilution buffer and 1 µl 20mg ml^–1^ proteinase K were added to 60 µl undiluted total protein. Then the input samples were processed in parallel with the immunoprecipitated samples from this point forward. 100 µl phenol:chloroform was added to the samples and the samples were centrifuged at 12 000rpm for 5min at 4 °C. Next, 10 µl 3M NaAc (pH 5.2), 6 µl 5mg ml^–1^ glycogen and 225ml ethanol were added to the supernatants; the samples were stored at –80 °C for 30min. The samples were centrifuged at 12 000rpm for 20min at 4 °C. The resulting pellet was washed with 500 µl 70% ethanol and then air-dried for 2min. Total RNA was dissolved with 5 µl RNase free water and reverse-transcribed as described above. For semi-quantitative PCR, 1 µl cDNA was used for per reaction. 15 µl PCR reactions were performed using Taq DNA polymerase for 30 cycles. The primers were as follows: *GA3ox1*-LP (5′-TTACAAGTGGACCCCTAAAGACG-3′) and *GA3ox1*-RP (5′- TACAG AATGGTTAGGAGGGTGGAG-3′); *GFP*-LP (5′- ATG GT GAGCA AGGGCG AGGAG-3′) and *GFP*-RP (5′- CGCC GATGGGGGTGTTCTGCTG-3′).

## Results

### The mRNA levels of GA20ox3 are regulated by *TSN*

**Fig. 1.**
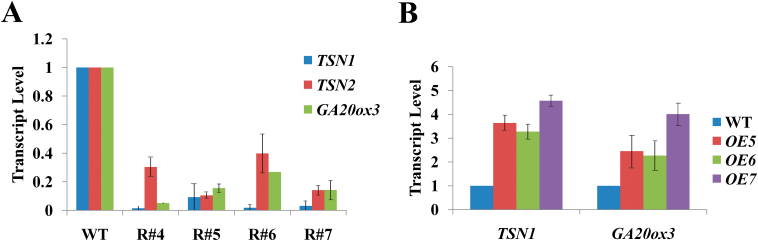
Expression of *TSN* and *GA20ox3* detected by quantitative RT-PCR. (A) Quantitative RT-PCR in *TSN1*/*TSN2* RNAi transgenic lines (R#4, R#5, R#6, R#7) and wild-type (WT) plants. (B) Quantitative RT-PCR in *TSN1* overexpression lines (*OE*5, *OE*6, *OE*7) and WT plants. Each value indicates relative quantities with the genes expressed in WT set at 1.0. Error bars indicate the standard error for the average of three independent experiments. (This figure is available in colour at *JXB* online.)

In previous studies, we found that the mRNA levels of *GA20ox3*, which encodes a key enzyme for GA biosynthesis, were decreased in a *tsn1* (named *tudor2*) mutant (Liu *et al*., 2010). To further confirm that the expression of *GA20ox3* is regulated by TSN, we constructed a pFGC-*TSN1*/*TSN2* RNAi vector and transformed it into Columbia type *Arabidopsis*. We selected the homozygous transgenic lines to investigate the mRNA levels of *TSN* and *GA20ox3* by semi-quantitative RT-PCR (Supplementary Fig. S1, available at JXB online) and quantitative RT-PCR (Fig. 1). As shown in Fig. 1A, four lines (#4–#7) demonstrated decreased *TSN1* and *TSN2* expression. The mRNA levels of *GA20ox3* were also down-regulated in these *TSN1*/*TSN2* RNAi transgenic lines. However, the total transcript amount of *TSN1* plus *TSN2* was not completely correlated to the transcript amount of *GA20ox3* in the RNAi lines (Fig. 1A). This may be because TSN1 and TSN2 play unequal roles in regulating *GA20ox3* levels. Dit Frey *et al*. (2010) reported that TSN1 and TSN2 are functionally redundant, but TSN1 shows moderate predominance. We also overexpressed *TSN1* cDNA driven by a 35S promoter in WT plants. We selected the homozygous transgenic T2 lines to investigate the mRNA levels of *TSN1* and *GA20ox3.* The mRNA levels of *TSN1* increased by varying degrees in all transgenic lines. The *GA20ox3* mRNA levels also increased accordingly (Fig. 1B; Supplementary Fig. S1B, available at JXB online). We observed a strong correlation between *TSN1* transcript amount and *GA20ox3* transcript amount.

### *TSN1* OE lines display the phenotypes of GA overproduction, but *TSN1/TSN2* RNAi lines do not show obvious defects under normal conditions

**Fig. 2.**
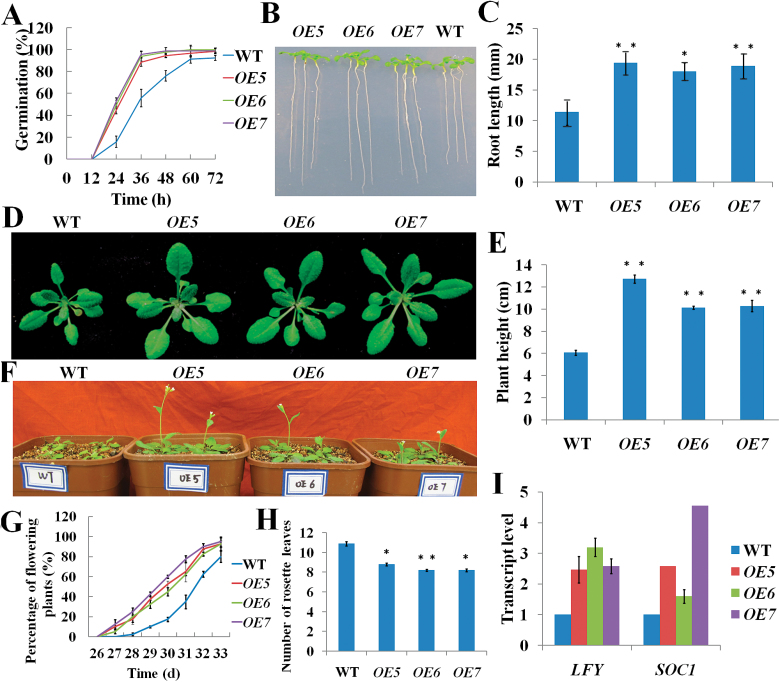
Phenotype characterization of wild-type (WT) plants and *TSN1* overexpression (OE) lines (*OE*5, *OE*6, *OE*7). (A) Germination on MS medium. (B) Phenotype of 7-day-old seedlings. (C) Root length of 7-day-old seedlings. (D) Phenotype of 24-day-old plants. (E) Stem length of 40-day-old plants. (F) Flowering phenotype. (G) Percentage of flowering plants (40 plants per line). (H) Number of rosette leaves from the primary bolt to reach a height of 1cm for each OE and WT plants (40 plants per line). (I) Levels of floral integrator gene (*LFY*, *SOC1*) transcripts. RNA was isolated from 8-day-old seedlings for quantitative RT-PCR. Each value indicates relative quantities with the genes expressed in WT set at 1.0. Error bars indicate the standard error for the average of three independent experiments. *P<0.05 and **P<0.01 (Student’s t-test) indicate significant differences between OE and WT plants. (This figure is available in colour at *JXB* online.)

We selected three homozygous T3 OE transgenic lines (*OE*5, 6, and 7) for analysis of growth phenotypes. Under normal growth conditions, seed germination in the OE lines was completed sooner than in WT plants (Fig. 2A). Primary root length was longer in OE plants than WT plants (Fig. 2B, C), and leaf size and stem length were also increased in OE plants (Fig. 2D, E). All three OE lines flowered earlier than WT plants (Fig. 2F–H). SUPPRESSOR OF OVEREXPRESSION OF CO1 (SOC1) and LEAFY (LFY) are floral integrators during the floral transition in *Arabidopsis* (Blázquez and Weigel, 2000; Simpson and Dean, 2002). GA promotes *SOC1* expression and SOC1 promotes *LFY* expression (Moon *et al.*, 2003; Lee *et al.*, 2008). Therefore, we detected levels of both floral integrator gene transcripts. We found that *LFY* and *SOC1* transcript levels increased in OE lines (Fig. 2I). All of the phenotypes were similar to those of the *GA20ox* overexpressed transgenic lines (Huang *et al*., 1998; Coles *et al*., 1999), which suggests that the phenotypes of the *TSN1* OE lines are primarily caused by increased *GA20ox3* mRNA levels. However, RNAi transgenic lines did not show obvious defects under normal growth conditions (Table 1; Supplementary Fig. S2 ,available at JXB online). This may be because *GA20ox3* demonstrates partial functional redundancy with *GA20ox1* and *GA20ox2* under normal conditions (Plackett *et al.*, 2012).

**Table 1.** Fresh weights of 17-day-old wild-type (WT), *ga20ox3, TSN1/TSN2* RNAi (R#4, R#7), and *TSN1* overexpression (*OE*5, *OE*7) seedlings grown on MS medium or MS medium containing 150mM NaCl or 200mM mannitol (n=30 for each line)Values are reported as mg fresh weight. Data represent the mean±SD of three independent biological determinations. *P<0.05 and **P<0.01 (Student’s t-test) indicate significant differences between mutants or transgenic lines and WT plants.

Lines	*ga20ox3-1*	*ga20ox3-2*	R#4	R#7	WT	*OE*5	*OE*7
MS	130.6±6.8	137.0±5.3	132.1±3.1	126.4±5.3	132.8±4.1	170.3±2.8^**^	168.9±4.3^**^
NaCl	60.5±2.7^**^	56.5±2.8^**^	48.7±3.1^**^	60.4±2.5^**^	82.3±7.5	102.3±5.6^*^	128±5.0^**^
Mannitol	63.2±1.8^**^	66.1±2.9^**^	75.1±1.9^**^	50.4±1.1^**^	81.7±2.8	100.8±3.4^**^	98.8±3.8^**^

### The growth of *ga20ox3* mutants and *TSN1/TSN2* RNAi transgenic lines are impaired under salt stress, whereas TSN1 OE transgenic lines are more resistant to stress

**Fig. 3.**
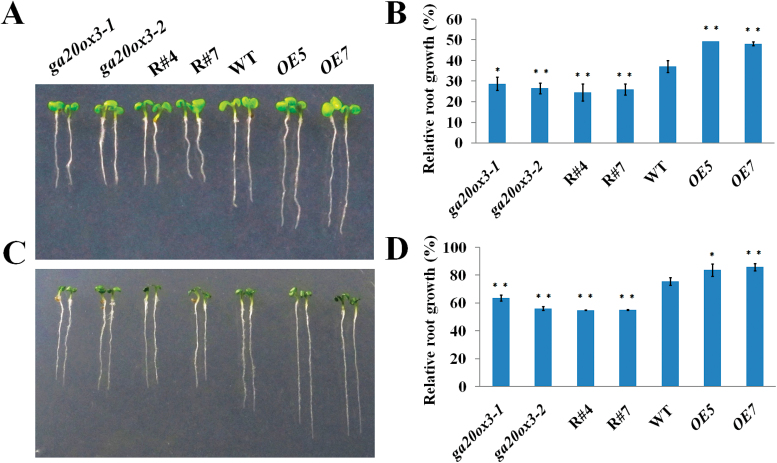
Phenotype characterization of wild-type (WT), *ga20ox3*, *TSN1*/*TSN2* RNAi (R#4, R#7), and *TSN1* overexpression (*OE*5, *OE*7) seedlings under stress conditions. (A, C) Phenotype of seedlings treated with 150mM NaCl (A) and 200mM mannitol (C). (B, D) Relative root growth of seedlings treated with 150mM NaCl (B) and 200mM mannitol (D). The seeds were germinated for 2 d under normal growth conditions and then treated with 150mM NaCl or 200mM mannitol. The primary roots of 6-day-old seedlings were measured and the relative growth is reported as the mean length. *P<0.05 and **P<0.01 (Student’s t-test) indicate significant differences between mutants or transgenic lines and WT plants. Error bars indicate the standard error for the average of three independent experiments. (This figure is available in colour at *JXB* online.)

Dit Frey *et al*. (2010) reported that TSN is necessary for stress tolerance in *Arabidopsis*. Therefore, we performed stress experiments to observe the phenotypes of RNAi and OE lines. Seeds from the transgenic lines, as well as WT plants, were germinated on MS medium for 2 d and then transferred to MS medium containing various concentrations of NaCl (50, 100, 150, and 200mM) or mannitol (100, 200, and 300mM). The most obvious phenotypic differences between transgenic lines and WT seedlings were observed with 150mM NaCl and 200mM mannitol. Therefore, we selected 150mM NaCl and 200mM mannitol for performing further experimental analyses. As shown in Fig. 3, compared with WT seedlings, the growth of RNAi lines was more severely affected when the seedlings were grown on MS medium containing 150mM NaCl or 200mM mannitol; conversely, OE transgenic lines were more resistant to NaCl stress (Fig. 3A, B) and mannitol stress (Fig. 3C, D) than WT plants. To investigate whether the defective phenotypes of RNAi lines were caused by decreased expression of *GA20ox3*, we performed experiments under stress to observe the phenotypes of the two mutants of *GA20ox3* (*ga20ox3-1* and *ga20ox3-2*). In the *ga20ox3-1* mutant, C-493 is replaced by T, which generated a premature stop codon and resulted in the complete loss of GA20ox activity *in vitro* (Plackett *et al.*, 2012). In the *ga20ox3-2* mutant, C-357 is replaced by T, which led to Pro-119 being replaced by Ser. Neither of the *ga20ox3* mutants showed obvious defects under normal growth conditions (Plackett *et al.*, 2012; Table 1; Supplementary Fig. S2, available at JXB online). After germinating for 2 d, the seeds of the mutants were transferred to MS medium containing 150mM NaCl or 200mM mannitol. The growth of the two *ga20ox3* mutants was more severely inhibited than WT plants, which was similar to the growth in the RNAi transgenic lines (Fig. 3). The biomass accumulation decreased in *ga20ox3* mutants and RNAi lines and increased in OE lines (Table 1).

### The defective phenotypes of *ga20ox3* mutants and *TSN1*/*TSN2* RNAi transgenic lines can be rescued by GA_3_

**Fig. 4.**
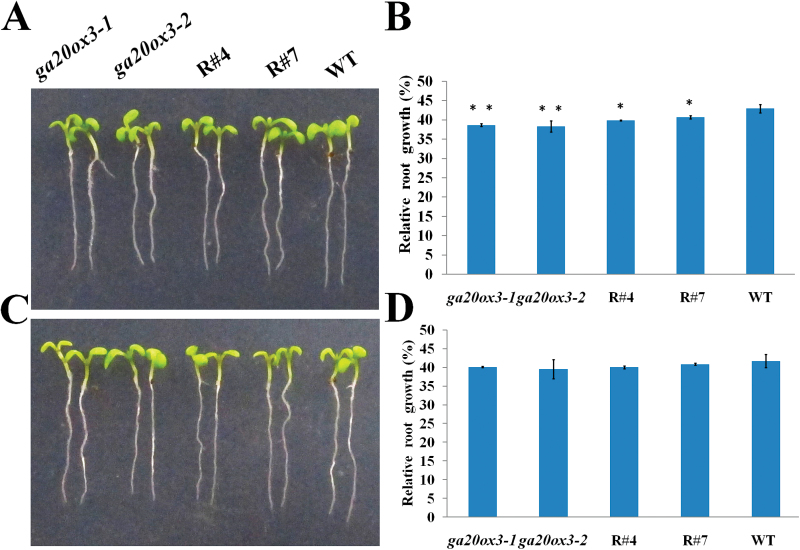
Phenotype characterization of wild-type (WT), *ga20ox3*, *TSN1*/*TSN2* RNAi (R#4, R#7) seedlings under salt stress conditions in response to GA_3_. (A, C) Phenotype of seedlings treated with 10 µM GA_3_ (A) and 100 µM GA_3_ (C) under 150mM NaCl stress. (B, D) Relative root growth of seedlings treated with 10 µM GA_3_ (B) and 100 µM GA_3_ (D) under 150mM NaCl stress. The seeds were germinated for 2 d under normal growth conditions and then transferred to MS medium supplemented with 150mM NaCl plus 10 µM GA_3_ and 100 µM GA_3_. The primary roots of 6-day-old seedlings were measured and the relative growth is reported as the mean length. *P<0.05 and **P<0.01 (Student’s t-test) indicate significant differences between mutants or transgenic lines and WT plants. Error bars indicate the standard error for the average of three independent experiments. (This figure is available in colour at *JXB* online.)

We performed GA response experiments to test whether the defective growth phenotype of *TSN1*/*TSN2* RNAi transgenic lines was caused by the decreased expression of *GA20ox3*. The seeds of *ga20ox3* mutants and RNAi transgenic lines were germinated on MS medium for 2 d. The synchronized growth seedlings were then transferred to MS medium containing 150mM NaCl plus 10 µM or 100 µM GA_3_ and grown for 6 d. As shown in Fig. 4A, B and Table 2, treatment with 10 µM GA_3_ was able to partly restore the defective growth of *ga20ox3* mutants and RNAi transgenic lines. Moreover, the growth of mutants almost completely reverted to the level of WT plants when treated with 100 µM GA_3_ (Fig. 4C, D; Table 2). These results indicate that exogenous GA_3_ can rescue the defective phenotypes of mutant plants under salt stress.

**Table 2.** Fresh weights of 8-day-old wild-type (WT), *ga20ox3*, and *TSN1/TSN2* RNAi (R#4, R#7) seedlings grown on MS medium containing 150mM NaCl or 150mM NaCl plus 10 µM GA_3_ or 100 µM GA_3_ (*n*=30 for each line)Values are reported as mg fresh weight. Data represent the mean±SD of three independent biological determinations. *P<0.05 and **P<0.01 (Student’s t-test) indicate significant differences between mutants or transgenic lines and WT plants.

Lines	*ga20ox3-1*	*ga20ox3-2*	R#4	R#7	WT
NaCl	18.6±2.3^**^	19.8±1.0^**^	22.2±3.7^**^	20.5±1.0^**^	25.7±0.9
NaCl+10 µM GA_3_	26.8±6.6	27.9±2.0^*^	26.1±0.5^*^	27.6±3.3	29.3±3.0
NaCl+100 µM GA_3_	31.9±3.4	33.6±2.0	32.3±2.6	32.6±2.5	33.3±3.5

### *GA20ox3* is highly expressed under salt stress

**Fig. 5.**
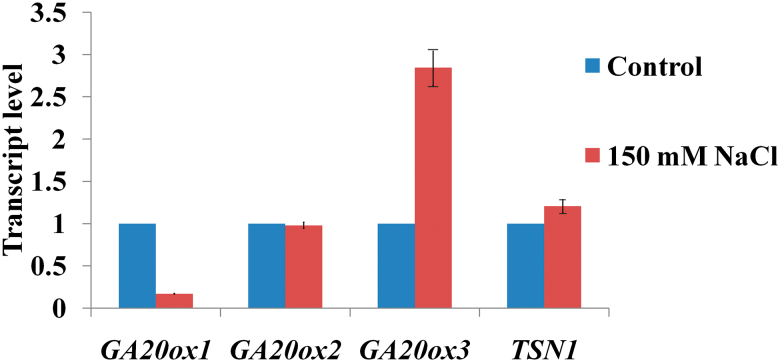
Expression of *GA20ox1*, *GA20ox2*, *GA20ox3*, and *TSN1* in wild-type seeds that were imbibed in 150mM NaCl solution or water for 24h. Expression levels were detected by quantitative RT-PCR. Each value indicates relative quantities with genes expressed in seeds treated with water set at 1.0. Error bars indicate the standard error for the average of three independent experiments. (This figure is available in colour at *JXB* online.)

As *ga20ox3* mutants display severe growth defects under stress conditions, we examined the expression of *GA20ox* under salt stress. The mRNA levels of *GA20ox3* were up-regulated in seeds imbibed with 150mM NaCl solution and *GA20ox1* expression decreased markedly. *GA20ox2* expression showed no obvious changes compared with the control (Fig. 5). We also examined *TSN1* transcript levels under stress. No obvious changes in mRNA levels were observed in response to NaCl treatment (Fig. 5), which suggests that salt stress may affect TSN1 protein levels or activity instead of mRNA levels.

### TSN1 accumulates to higher levels and locates to small cytoplasmic granules in response to salt stress

**Fig. 6.**
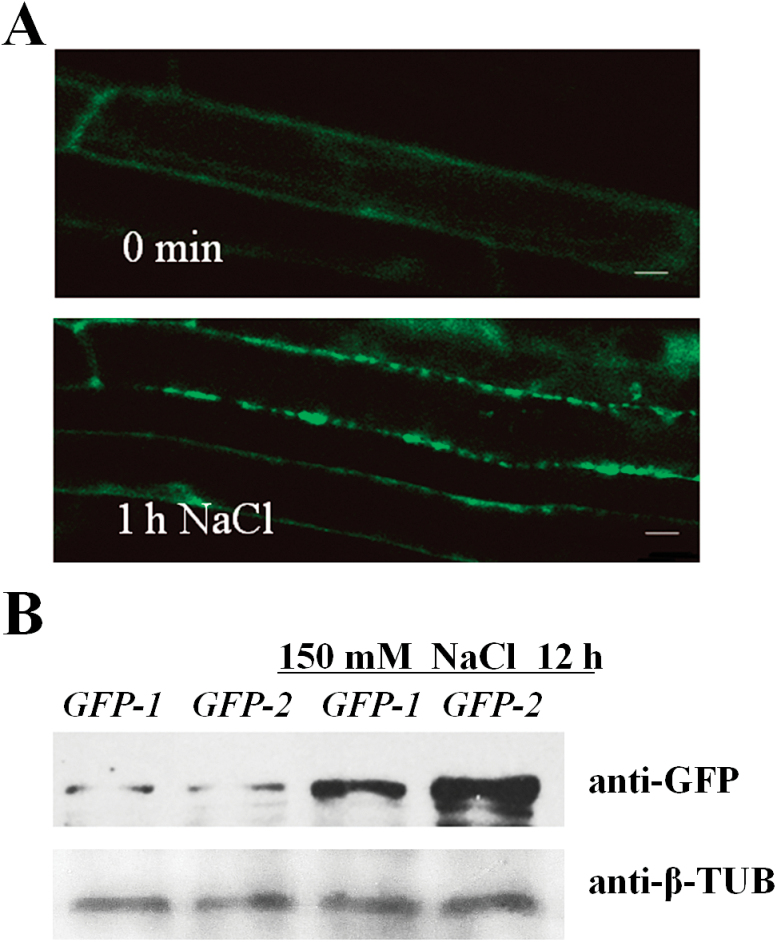
Subcellular localization of the TSN1–GFP protein. TSN1 accumulated to higher levels under salt stress. (A) TSN1–GFP in the primary roots of 6-day-old TSN1–GFP seedlings was localized uniformly in the cytoplasm under normal conditions (upper), but was redistributed to small granules after 1h of treatment with 150mM NaCl (lower). Scale bars: 10 µm. (B) TSN1–GFP was immunodetected by anti-GFP antibodies in a western blot analysis. Total protein was extracted from 7-day-old seedlings in TSN1–GFP transgenic lines (*GFP-1* and *GFP-2*) that were treated with 150mM NaCl for 12h or not treated (control). β-tubulin served as the sample-loading control. (This figure is available in colour at *JXB* online.)

We constructed the pBI121–*TSN1*–GFP vector and transformed the construct into WT plants to investigate the subcellular localization of TSN1. TSN1–GFP transgenic lines displayed phenotypes similar to that of OE transgenic lines, indicating that GFP-fusion don’t interfere with the biological function of TSN1. We found that the TSN1–GFP fusion protein was uniformly distributed in the cytoplasm under normal growth conditions (Fig. 6A, upper). We also investigated its subcellular localization under salt stress. Six-day-old TSN1–GFP transgenic seedlings were transferred to MS medium containing 150mM NaCl. TSN1 was markedly redistributed within an hour of imposition of NaCl stress, and small granules were rapidly formed in the cytoplasm (Fig. 6A, lower). Furthermore, we noted enhanced GFP fluorescence in the root of the NaCl-treated seedlings compared with the control group (no NaCl treatment; Fig. 6A). We performed a western blot analysis to test whether TSN1 levels increased in response to salt stress. We used affinity-purified GFP antibodies and detected higher levels of TSN1–GFP accumulation in TSN1–GFP transgenic seedlings treated with NaCl for 12h than in control seedlings (Fig. 6B).

### TSN1 co-localizes with RBP47 under stress

**Fig. 7.**
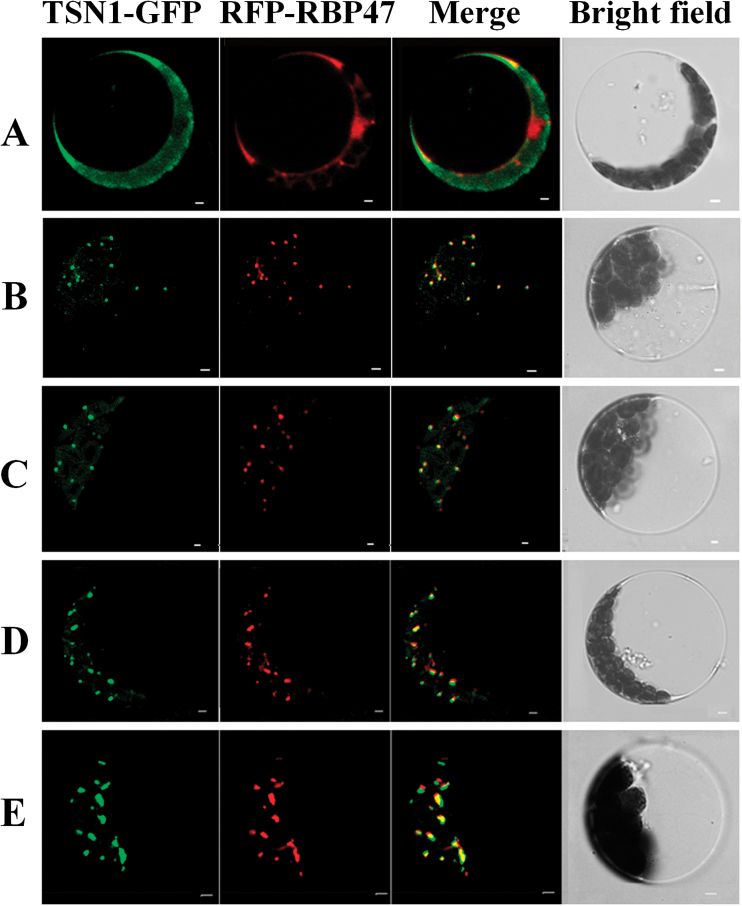
TSN1–GFP was co-localized with RFP–RBP47 in the SGs under salt stress conditions. (A) The protoplasts that were transformed with plasmids of TSN1–GFP and RFP–RBP47 were incubated under normal conditions. (B–E) The protoplasts that were transformed with plasmids of TSN1–GFP and RFP–RBP47 were treated with 150mM NaCl stress for 0.5h (B), 1h (C), 2h (D), and 3h (E). Scale bars: 2 µm. (This figure is available in colour at *JXB* online.)

A large number of small granules formed in the cytoplasm in TSN1–GFP transgenic lines under salt stress. This prompted us to investigate whether these granules were SGs that assembled in response to stress. RBP47 is the homologue of the mammals TIA-1 and is a marker protein for SGs in response to stress in *Arabidopsis* (Weber *et al.*, 2008). We performed the TSN1–GFP and RFP–RBP47 co-expression experiment under salt stress. Plasmids of TSN1–GFP and RFP–RBP47 were transiently co-expressed in living *Arabidopsis* protoplasts. Initially, RFP–RBP47 was primarily located in the nucleus (Fig. 7A), but it relocated to cytoplasmic granules in response to 150mM NaCl treatment (Fig. 7B–E). The granule size increased from 0.5 µm to 3 µm with increasing time under stress. This location and distribution of RFP–RBP47 is consistent with the results observed under heat stress by Weber *et al*. (2008). TSN1–GFP was dispersed in the cytoplasm under control conditions (Fig. 7A), but it aggregated into cytoplasmic granules in response to salt stress. TSN1–GFP fluorescence overlapped with RFP–RBP47 fluorescence (Fig. 7B–E). These results indicated that TSN1 accumulates in SGs under salt stress.

### TSN1 binds *GA20ox3* mRNA *in vivo*

**Fig. 8.**
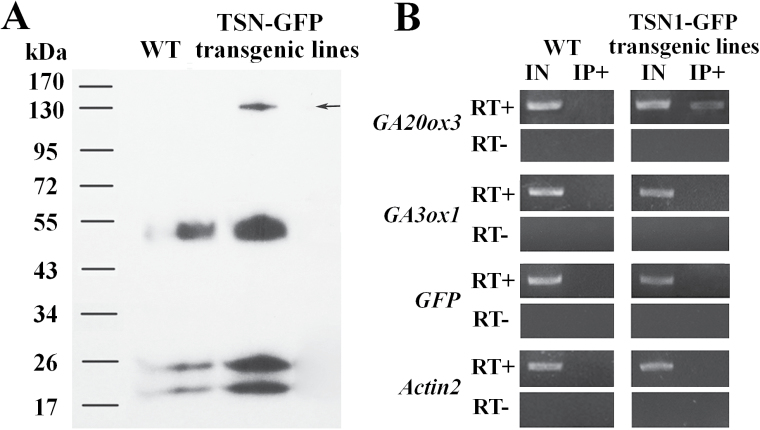
TSN1 binds *GA20ox3* mRNA *in vivo*. (A) Co-immunoprecipitation analysis indicated that TSN1–GFP was immunodetected by anti-GFP antibodies in TSN1–GFP transgenic lines but not in wild-type (WT) plants. The arrow indicates the TSN1–GFP fusion protein (135kDa in size). (B) RT-PCR of RNA isolated from immunoprecipitated samples (IP+) and input samples (IN). After reverse transcription, the cDNA was subjected to PCR using primers of *GA20ox3*, *GA3ox1*, *GFP*, and *Actin2* (RT+). *GA20ox3* mRNA could be specifically immunoprecipitated in TSN1–GFP transgenic lines (RT+). Genomic DNA contamination was monitored using mock reverse transcription (RT–).

TSN has been identified as an RNA-binding protein in rice (Sami-Subbu *et al.*, 2001) and our results showed that TSN is a component of SGs and modulates the mRNA levels of *GA20ox3*. Therefore, we performed RIP experiments to investigate the possibility that TSN interacts with *GA20ox3* mRNA. To ensure specificity of our RIP assays, we first performed co-immunoprecipitation using TSN1–GFP lines and affinity-purified anti-GFP antibodies. The result indicated that the TSN1–GFP was specifically immunoprecipitated by anti-GFP antibodies. A prominent high molecular weight band of 135kDa was readily evident in TSN1–GFP transgenic plants, but not in WT plants; this is the predicted size of the TSN1–GFP fusion protein (Fig. 8A). Other polypeptide bands observed at approximately 52, 25, and 20kDa were non-specific bands caused by IgG heavy and light chains. Purified RNA was detected by RT-PCR using *GA20ox3* primers. As shown in Fig. 8B, a band equivalent to the predicted size of the *GA20ox3* fragment was reproducibly co-immunoprecipitated using anti-GFP antibodies from TSN1–GFP transgenic plants. In contrast, no enrichment of *GA20ox3* was detected in RT(–), in which genomic DNA contamination was monitored using mock reverse transcription. The nucleotide acid sequence alignment showed that the RT-PCR products were indeed *GA20ox3* mRNA. This result indicates that TSN1 interacts with *GA20ox3* mRNA *in vivo*. To detect whether the interaction of *TSN1* and *GA20ox3* mRNA was specific, we assayed other mRNAs in the RNA precipitate. Although highly abundant in the input samples, *GA3ox1*, which encodes a key enzyme for GA biosynthesis, was not detected in immunoprecipitated samples (Fig. 8B). Likewise, abundant *GFP* and *Actin2* mRNAs were not co-precipitated in the RIP assays (Fig. 8B). These results indicate that TSN1 binds *GA20ox3* mRNA specifically *in vivo*.

## Discussion

### TSN regulates growth under stress by modulating GA20ox3 in *Arabidopsis*

TSN is a ubiquitous protein that is highly conserved in eukaryotes. In animal cells, TSN reportedly activates transcription and subsequent mRNA splicing and regulates RNA silencing (Tong *et al.*, 1995; Leverson *et al.*, 1998; Caudy *et al.*, 2003). Dit Frey *et al*. (2010) showed that TSN is essential for stress tolerance in plants. Our previous study showed that the mRNA levels of *GA20ox3*, which encodes a key enzyme for GA biosynthesis, was decreased in a *TSN1* T-DNA insertion mutant (Liu *et al*., 2010). These findings prompted us to investigate whether TSN functions in stress adaptation by regulating *GA20ox3*. To address this question, we first obtained constitutively expressed *TSN1*/*TSN2* RNAi transgenic lines and *TSN1* overexpression lines. As expected, the expression of the *GA20ox3* transcript was down-regulated in RNAi transgenic lines (Fig. 1A) and up-regulated in OE lines (Fig. 1B). The OE lines also displayed phenotypes of overproduction of GA (Fig. 2). RNAi lines did not show any obvious defects under normal growth conditions, which was in accordance with the phenotypes of double mutant *tsn1 tsn2* under non-stress conditions (Table 1; Supplementary Fig. S2; Dit Frey *et al*., 2010). When grown under salt-stress conditions, the RNAi lines displayed slower growth than WT plants, whereas *TSN1* OE lines displayed the opposite phenotypes (Fig. 3; Table 1). That is, the OE lines were more resistant to stress. We also performed phenotype analyses of two *GA20ox3* mutants, *ga20ox3-1* and *ga20ox3-2.* Neither of the mutants showed any defective phenotypes under normal conditions (Table 1; Supplementary Fig. S2, available at JXB online), but they displayed slower growth phenotypes similar to the *TSN1*/*TSN2* RNAi lines under salt stress (Fig. 3; Table 1). To test whether the defective growth phenotypes of these mutants were caused by the decreased expression of *GA20ox3*, we performed GA response experiments. The root length and biomass accumulation of mutants almost completely reverted to the level of WT plants when treated with 100 µM GA_3_ (Fig. 4; Table 2). Furthermore, through RIP experiments, we showed that TSN1 bound *GA20ox3* mRNA *in vivo* (Fig. 8). All of these results indicate that TSN regulates *Arabidopsis* growth under salt stress, at least partially, by modulating levels of *GA20ox3* mRNA.

### GA20ox3 is required to maintain plant growth under salt stress

GAs are well-known phytohormones and a family of tetracyclic diterpenoid carboxylic acids that promote cell division and elongation in several tissues and regulate multiple processes of plant development, such as seed germination, vegetative growth, flowering and fruit development. GA biosynthesis is primarily controlled by homeostatic mechanisms based on the negative feedback regulation of *GA20ox* and *GA3ox*, both of which are involved in the synthesis of bioactive GAs, and the activation of the GA 2-oxidases, which convert bioactive GAs to inactive forms (Thomas and Hedden, 2006; Rieu *et al*., 2008b; Yamaguchi, 2008). These enzymes occur in small gene families in *Arabidopsis* and have tissue-specific expression patterns that allow for precise spatial and temporal control of GA levels (Mitchum *et al.*, 2006; Rieu *et al*., 2008a). The environmental stimuli are significant factors that control the expression of these dioxygenase genes (Wu *et al*., 1996; Xu *et al*., 1997; Jackson *et al*., 2000; Yamauchi *et al.*, 2004; Colebrook *et al*., 2014). It was reported that abiotic stresses such as salt reduced bioactive GA levels (Achard *et al.*, 2006; Magome *et al*., 2008) by up-regulation of *GA2ox* genes, particularly *GA2ox7* (Magome *et al*., 2008). Magome *et al*. (2008) also found that *GA20ox1* were unregulated under salt stress. However, we observed that *GA20ox1* decreased markedly in seeds imbibed with 150mM NaCl solution, and *GA20ox3* transcript levels were up-regulated in response to salt stress (Fig. 5). This may reflect differences in tissues or salt concentrations used in the two experiments. Magome *et al*. (2008) used 2-week-old seedlings treated with 250mM NaCl, whereas we used seeds imbibed with 150mM NaCl solution. Furthermore, we observed that two *ga20ox3* mutants grew slower than WT plants under 150mM NaCl stress (Fig. 3; Table 1), but no defects were observed under normal growth conditions (Table 1; Supplementary Fig. S2). On the basis of these findings, we conclude that *GA20ox3* is required to maintain plant growth under salt stress. Recently, Sun *et al.* (2013) reported that a GA-induced protein, GASA14, promoted growth and enhanced abiotic stress resistance. Possibly, GA20ox3 catalyses GAs biosynthesis under stress and GAs function via GASA’s pathway to maintain plant growth under stress.

### As a component of SGs, TSN may be required for optimal translation *GA20ox3* mRNA under salt stress

Regulating mRNA stability and translation activity allows a cell to rapidly alter the proteome in response to various signals. SGs are dynamic, dense structures that rapidly form in the cytoplasm in response to stress stimuli. In mammals, SG formation is thought to be involved in stability regulation of mRNA and therefore required for optimal translation of stress-responsive mRNAs (Buchan and Parker, 2009; Vanderweyde *et al*., 2013). For example, ZBP1, an RNA-binding protein, is concentrated in SGs bound to its associated mRNAs and enhances the stability of these mRNAs under stress (Stöhr *et al*., 2006). CUGBP1, a member of the CELF family of RNA binding proteins, accumulates in SGs together with its associated *p21* mRNA and enhances the stability and expression level of *p21* mRNA upon bortezomib treatment (Gareau *et al*., 2011).

Although SG assembly and function has been investigated extensively in mammals, there have been limited investigations in plants. Weber *et al*. (2008) showed that plants contain SGs that are conserved from mammals. We showed that TSN1 is a novel component of plant SGs through fluorescence co-localization experiments (Fig. 7) and TSN1 interacts with *GA20ox3* mRNA *in vivo* (Fig. 8). These results suggest that *GA20ox3* mRNA is recruited into SGs by binding with TSN. We also showed that TSN modulates *GA20ox3* mRNA levels (Fig. 1) and *GA20ox3* mRNA levels are increased under salt stress (Fig. 5). Thus, we speculated TSN concentrated in SGs bound to *GA20ox3* mRNAs enhances the stability of *GA20ox3* mRNA and allow its optimal translation to regulate plant growth under salt stress. However, the detailed mechanism by which SGs regulates *GA20ox3* mRNA needs to be further investigated.

## Supplementary data

Supplementary data are available at *JXB* online

Figure S1. Expression of *TSN* and *GA20ox3* detected by semi-quantitative RT-PCR in *TSN1*/*TSN2* RNAi transgenic lines and *TSN1* overexpression lines

Figure S2. Phenotypes of 7-day-old seedlings of *ga20ox3* mutants, *TSN1*/*TSN2* RNAi transgenic lines and WT plants under normal conditions

Supplementary Data
