# Supplementary material for: Tudor-SN, a component of stress granules, regulates growth under salt stress by modulating GA20ox3 mRNA levels in Arabidopsis
Source: J Exp Bot. 2014 Sep 9;65(20):5933–44. doi: 10.1093/jxb/eru334 (PMC4203129; doi:10.1093/jxb/eru334)
Supplement: Supplementary Data [file supp_65_20_5933__index.html]

Tudor-SN, a component of stress granules, regulates growth under salt stress by modulating GA20ox3 mRNA levels in Arabidopsis — Tudor-SN, a component of stress granules, regulates growth under salt stress by modulating GA20ox3 mRNA levels in Arabidopsis — Supplementary Data 

# Tudor-SN, a component of stress granules, regulates growth under salt stress by modulating *GA20ox3* mRNA levels in *Arabidopsis*

## Supplementary Data

Data files

**Files in this Data Supplement:**

- Supplementary Data - Supplementary Data
