# Supplementary material for: Tudor-SN, a component of stress granules, regulates growth under salt stress by modulating GA20ox3 mRNA levels in Arabidopsis
Source: J Exp Bot. 2014 Sep 9;65(20):5933–44. doi: 10.1093/jxb/eru334 (PMC4203129; doi:10.1093/jxb/eru334)
Supplement: Supplementary Data [file supp_eru334_jexbot127704_file001.pdf]

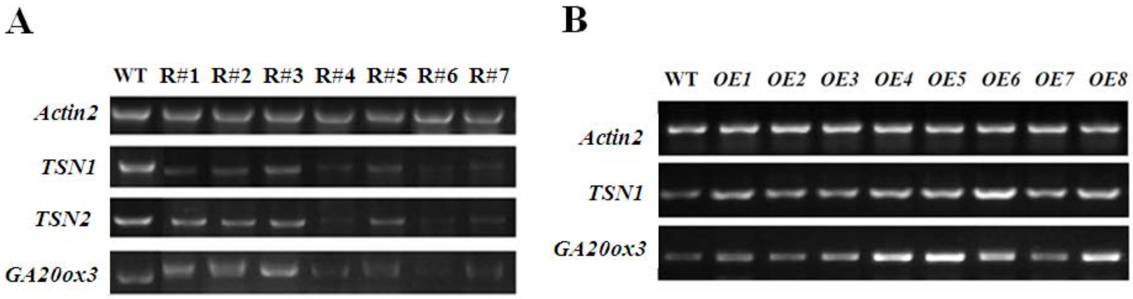

Fig. S1. Expression of *TSN* and *GA20ox3* detected by semi-quantitative RT-PCR. (A) Semi-quantitative RT-PCR in *TSN1/TSN2* RNAi transgenic lines. (B) Semi-quantitative RT-PCR in *TSN1 OE* lines. *Actin2* was used as the control of semi-quantitative RT-PCR.

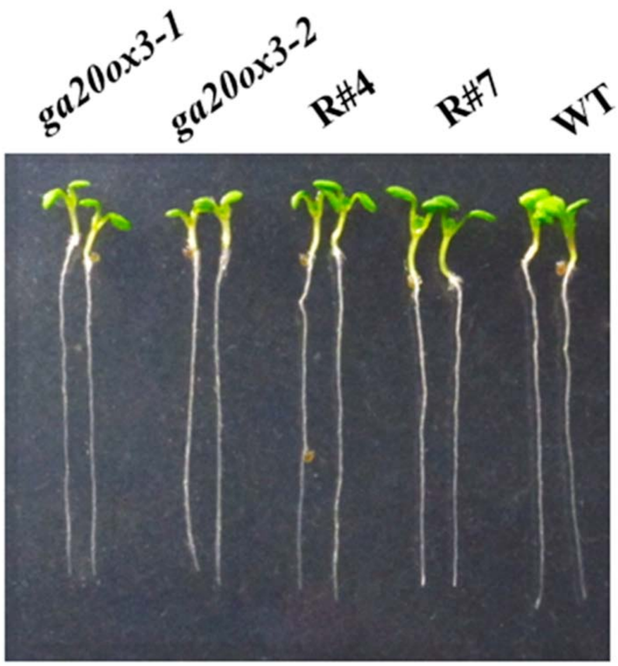

Fig. S2. Seven-day-old seedings of *ga20ox3*, *TSN1/TSN2* RNAi and WT under normal condition.
